# PESM: predicting the essentiality of miRNAs based on gradient boosting machines and sequences

**DOI:** 10.1186/s12859-020-3426-9

**Published:** 2020-03-18

**Authors:** Cheng Yan, Fang-Xiang Wu, Jianxin Wang, Guihua Duan

**Affiliations:** 1 Hunan Provincial Key Lab on Bioinformtics, School of Computer Science and Engineering, Central South University, 932 South Lushan Rd, ChangSha, 410083 China; 2 School of Computer and Information,Qiannan Normal University for Nationalities, Longshan Road, DuYun, 558000 China; 3 Biomedical Engineering and Department of Mechanical Engineering, University of Saskatchewan, Saskatoon, SKS7N5A9 Canada

**Keywords:** MiRNA, Essentiality, Gradient boosting machines

## Abstract

**Background:**

MicroRNAs (miRNAs) are a kind of small noncoding RNA molecules that are direct posttranscriptional regulations of mRNA targets. Studies have indicated that miRNAs play key roles in complex diseases by taking part in many biological processes, such as cell growth, cell death and so on. Therefore, in order to improve the effectiveness of disease diagnosis and treatment, it is appealing to develop advanced computational methods for predicting the essentiality of miRNAs.

**Result:**

In this study, we propose a method (PESM) to predict the miRNA essentiality based on gradient boosting machines and miRNA sequences. First, PESM extracts the sequence and structural features of miRNAs. Then it uses gradient boosting machines to predict the essentiality of miRNAs. We conduct the 5-fold cross-validation to assess the prediction performance of our method. The area under the receiver operating characteristic curve (AUC), F-measure and accuracy (ACC) are used as the metrics to evaluate the prediction performance. We also compare PESM with other three competing methods which include miES, Gaussian Naive Bayes and Support Vector Machine.

**Conclusion:**

The results of experiments show that PESM achieves the better prediction performance (AUC: 0.9117, F-measure: 0.8572, ACC: 0.8516) than other three computing methods. In addition, the relative importance of all features also further shows that newly added features can be helpful to improve the prediction performance of methods.

## Background

MicroRNAs (miRNAs) are small non-coding RNAs with a length of 22 nucleotides, which are processed from stem-loop regions of longer RNA transcripts [1]. They bind to the 3’ untranslated regions (UTRs) of target mRNAs by sequence-specific base pairing to regulate the gene expression at the post-transcriptional level [2, 3]. Studies have shown that miRNAs play crucial roles in many biological processes, such as cell differentiation, growth, immune reaction and death, thereby leading to a variety of diseases [4, 5]. For example, miR-28-5p and miR-28-3p are down-regulated in colorectal cancer (CRC) samples compared with normal colon samples [6]. Members of the let-7 family of microRNAs were significantly downregulated in primary melanomas, and the anchorage-independent growth of melanoma cells are also inhibited by let-7b [7]. The poor clinical features in gastric cancer are associated with the low levels of miR-34b and miR-129 expression [8]. The incidence of lymphoma is regulated by the overexpression of miRNA hsa-mir-451a [9, 10]. Furthermore, after knocking out one or more members of a very broadly conserved miRNA family, some abnormal phenotypes are observed [11]. For example, as paralogous proteins, members of the same seed families often have at least partially redundant functions, with severe loss-of-function phenotypes apparent only after multiple family members are disrupted, which includes mmu-mir-22 [12], mmu-mir-29 [13].

In order to systematically understand the associated mechanisms between miRNAs and diseases, some databases have been constructed, such as HMDD [14], miR2Disease [15], dbDEMC [16], Oncomirdb [17]. With these databases, some computational methods have been proposed to identify potential miRNA-disease associations. Based on a kernelized Bayesian matrix factorization model, Lan et al. proposed a computational method (KBMF-MDI) to predict miRNA-diseases associations based on known miRNA-disease associations, miRNA sequence and disease sematic information [18]. By integrating the miRNA-disease association network, miRNA similarity network and disease similarity network, You et al. [19] developed PBMDA to prioritize the underlying miRNA-disease associations, which used a special depth-first search algorithm in a heterogeneous network. Luo et al. also proposed a network-based method for drug repositioning based on similarities among drugs and diseases [20]. DNRLMF-MDA was proposed to discover hidden miRNA-disease associations based on known miRNA-disease associations, miRNA similarity and disease similarity, the main feature of DNRLMF-MDA was that it assigned higher importance levels to the observed interacting miRNA-disease pairs than unknown pairs [21]. Based on the inductive matrix completion model, IMCMDA was also proposed to predict miRNA-disease associations by integrating miRNA functional similarity, disease semantic similarity and Gaussian interaction profile kernel similarity [22]. Chen et al. [23] proposed a computational model named Laplacian regularized sparse subspace learning for miRNA-disease association prediction (LRSSLMDA), which projected miRNA/disease’ statistical feature profiles and graph theoretical feature profiles to a common subspace. MDHGI was a computational model to discover new miRNA-disease associations based on the matrix decomposition and heterogeneous graph inference, which integrated the predicted association probability obtained from matrix decomposition through a sparse learning method [24]. DLRMC was a computational method to predict miRNA-disease associations, based on matrix completion model with dual Laplacian regularization (DLRMC) [25]. EDTMDA was a computational method based on the ensemble of decision trees, which built a computational framework by integrating ensemble learning and dimensionality reduction [26]. Based on the logistic model tree, Wang et al. proposed a method for predicting miRNA-disease associations (LMTRDA) [27]. Pasquier et al. proposed a method to calculate the associations of miRNA disease pairs according to the vector similarity of miRNAs and diseases based on the distributional information of miRNAs and diseases in a high-dimensional vector space [28]. RKNNMDA was a type of instance-based learning to predict potential miRNA-disease associations based on the k-nearest neighbor algorithm and support vector machine (SVM) [29]. BNPMDA was a novel computational model of bipartite network projection for miRNA-disease association prediction, and its main feature was that bias ratings were constructed for miRNAs and diseases by using agglomerative hierarchical clustering [30]. VAEMDA was a novel miRNA-disease association prediction method based on an unsupervised deep learning framework with variational autoencoder [31]. Yan et al. proposed ABMDA to predict potential miRNA-disease associations, which balanced the positive and negative samples by performing random sampling based on k-means clustering on negative samples [32]. Based on the k-mer sparse matrix to extract miRNA sequence information and deep auto-encoder neural network (AE), MLMDA was developed to predict miRNA-disease associations [33]. Cheng et al. also proposed a miRNA-disease association prediction method based on adaptive multi-view multi-label learning(AMVML) [34]. By combined the weighted profile and collaborative matrix factorization (CMF), a new computation model logistic weighted profile-based collaborative matrix factorization (LWPCMF) was developed to predict miRNA-disease associations [35]. DBMDA was a novel computational model for miRNA-disease association prediction, the notable feature of this method was inferring the global similarity from region distances based on the miRNA sequences [36]. By combing the kernel-based nonlinear dimensionality reduction, matrix factorization and binary classification, a neoteric Bayesian model (KBMFMDA) was proposed to predict miRNA-disease associations [37]. Chen et al. also proposed a miRNA-disease association prediction method (NCMCMDA) based on a neighborhood constraint matrix completion model [38]. Based on the neural inductive matrix completion with graph convolutional networks, Li et al. also proposed a method to predict miRNA-disease associations [39]. In addition, the matrix completion model was also used in drug repositioning [40–43], predicting lncRNA-disease associations [44, 45] and microbe-disease associations [46].

Furthermore, the miRNA-target interaction was also predicted by miRTRS based on known miRNA-target interactions, miRNA sequences and gene sequences [47]. Bartel et al. [11] described the important biological functions identified for most of the broadly conserved miRNAs of mammals, and they also reviewed how metazoan miRNAs recognized and caused the repression of their targets. Studies demonstrated that some miRNA molecules were essential to the disease development [48]. Therefore, inspired by the bioinformatics development of the protein essentiality prediction [49, 50], Gao et al. first proposed a computational method (miES) based on machine learning and sequence features to identify the miRNA essentiality [51]. MiES used the miRNA sequences and a logistic regression model for performing miRNAome-wide search for essential miRNAs. In addition, miES further analyzed the miRNA conservation [52], miRNA expression dataset and miRNA disease spectrum width (DSW) [53] to understand the important basis for predicting the essentiality of miRNAs [54]. In addition, the sequence features also used in study of genome [55]. The frequencies of k-mers were also used in ARP to classify the reads into three categories [56]. In MultiMotifMaker, the position weight matrix (PWM) was a used representation of motifs, and its 4 columns (A,C,T,G) described the frequency of occurrence of each base at each position [57].

However, the current development of miRNA essentiality prediction method is still not good enough. Complex and deeper features related to miRNAs should be considered to improve the prediction quality of current methods. The more effective and advanced computational methods should also be developed to identify essential miRNAs. Therefore, in this study we propose a computational method (PESM) to predict potential essential miRNA based on the essential miRNA and non-essential miRNAs benchmark dataset. PESM first integrates more miRNA sequence features (such as 18 dinucleotide features : UC%, UG% and so on) as in miES. Then PESM uses gradient boosting machines to predict the essentiality of miRNAs. In order to assess the prediction performance of PESM and compare it with other computational methods, we also conduct the 5-fold cross validation (5CV). In addition, the area under of receiver operating characteristic (ROC) curve (AUC), accuracy (ACC) and F-measure are used as the metrics of all prediction methods. The competing methods include miES, Gaussian Naive Bayes (GaussianNB) and SVM. The experiment results of 5CV show that PESM can obtain better prediction performance in terms of AUC, ACC and F-measure (AUC: 0.9117, ACC: 0.8516 and F-mearsure: 0.8572) than other competing methods: miES (AUC: 0.8837, ACC: 0.8263 and F-mearsure: 0.8326), GaussianNB (AUC: 0. 8720, ACC: 0.8000 and F-mearsure: 0.8093) and SVM (AUC: 0.8571, ACC: 0.8206 and F-mearsure: 0.8271). Comparing with miES, PESM integrates more sequence and structural features of miRNAs. In addition, the gradient boosting machine model is used to compute the predicted scores of essential miRNAs. By analyzing the relative importance of the features, we can also conclude that the added new features can represent the intrinsical characteristics of miRNAs. Finally, the experiment results also prove that the prediction ability of our method is superior to other competing methods.

## Methods

### Materials

In this study, we use the benchmark dataset of essential miRNAs and non-essential miRNAs, which consists of the pre-miRNA sequences and mature-miRNA sequences of human, rat and mouse from miRbase [52]. The benchmark dataset includes 77 essential mice miRNAs and the same number of non-essential miRNAs [11]. The known essential mice miRNAs (positive samples) and non-essential miRNAs (negative samples) were obtained from the review paper [11]. In miES, the negative samples were generated with two strategies: (1) the random selection; (2) the selection according to the maximum mean AUC.

### Feature set

The miRNAs are transcribed as long primary miRNAs, which produce miRNA precursors (pre-miRNAs) by nuclear RNase III Drosha [58]. Then the pre-miRNAs are cleaved into mature miRNAs [1]. All pre-miRNAs have stem-loop hairpin structures [59]. Therefore, by considering the production process of miRNAs and the structure of pre-miRNAs, PESM uses the features of not only mature-miRNAs but also pre-miRNAs. The selected feature set of pre-miRNA sequences and mature-miRNA sequences has important influence on predicting the essentiality of miRNAs. In this study, we first extract the 14 pre-miRNA and mature-miRNA features which include information about sequences and structures. In addition, up to now various feature sets have been proposed to study pre-miRNA and other relative prediction problems. Inspired by the successful application of dinucleotide frequency information in predicting pre-miRNAs, we add the 18 dinucleotide frequency features of pre-miRNAs and mature-miRNAs in this study [60]. In addition, we further add other 6 structure features of pre-miRNAs, includes normalized base-pairing propensity (P(s)), normalized base-pairing propensity divided by its length (nP(s)), normalized Shannon entropy (Q(s)), normalized Shannon entropy divided by its length (nQ(s)), normalized base-pair distance(D(s)) and normalized base-pair distance divided by its length(nD(s)) [61]. We use the module RNAlib of Vienna RNA Package to intrinsic folding quantitative measures P(S), nP(S), Q(s), nQ(s), D(s) and nD(s) [62]. These structure features and Vienna RNA Package have been broadly used in both miRNA prediction and pre-miRNA prediction [63–65]. As a result, our method consists of 38 features. Note that these features also include the 14 features which are used in miES. The more detail about the feature set is described in Table 1.

**Table 1:** The feature set description

Category	Description	Number of features
Base content in pre-miRNAs	The content of base *S* in pre-miRNAs, *S*∈{*U*,*C*,*G*}	3
mature-miRNAs length	The sequence length of mature-miRNAs	1
Base content in mature-miRNAs	The content of base *S* in mature-miRNAs, *S*∈{*U*,*C*,*G*}	3
non-mature-miRNAs length	The sequence length of non-mature-miRNAs	1
Base content in non-mature-miRNAs	The content of base *S* in non-mature-miRNAs, *S*∈{*U*,*C*,*G*}	3
MFE and nMFE	The minimum free energy of pre-miRNA secondary structures and it is divided by its length	2
Cleavage site base class	The cleavage sites are assigned into 3 classes, 1: all cleavage sites of mature-miRNAs from the same pre-miRNAs are *U*; 0: not all cleavage sites are *U*; -1: all are non-*U*.	1
Dinucleotide pairs frequency in pre-miRNAs	The Dinucleotide pairs *SZ* frequency in pre-miRNAs, *S*,*Z*∈{*U*,*C*,*G*}	9
Dinucleotide pairs frequency in mature-miRNAs	The Dinucleotide pairs *SZ* frequency in mature-miRNAs, *S*,*Z*∈{*U*,*C*,*G*}	9
The structure feature of pre-miRNAs	Normalized base-pairing propensity (*P*(*s*)), Normalized base-pairing propensity divided by its length (*nP*(*s*)), Normalized Shannon entropy (*Q*(*s*)), Normalized Shannon entropy divided by its length (*nQ*(*s*)), Normalized base-pair distance (*D*(*s*)), Normalized base-pair distance divided by its length (*nD*(*s*))	6

### Gradient boosting regression trees

After computing the above sequence and structure features, we take a supervised learning method named gradient boosting regression trees derived from the gradient boosting machine model to predict essential miRNAs [66, 67]. This method has been successfully used in other classification issues [68, 69]. In the common supervised learning scenario, the sample data set can be represented by a set containing feature vectors and labels: *D*={(*x*_*i*_,*y*_*i*_)}(*i*=1,...,*N*), where *N* is the number of samples [70]. In this study, *x*_*i*_∈*R*^*d*^ is the feature vector of the *i*−*th* miRNA, while *y*_*i*_ is its essentiality score. *d* is the dimensionality of features. According to the gradient boosting regression tree model, the predicted essentiality score ${\hat y}_{i}$ of miRNA *i* from its input feature vector can be calculated as follows:

1 $$\begin{array}{@{}rcl@{}}  {\hat y}_{i} = \phi(x_{i}) = \sum\limits_{k=1}^{K}f_{k}(x_{i}),f_{k} \in F \end{array} $$

where *K* is the maximum depth of regression trees and *F* is a set of functions containing the partition of the region and score [70]. In order to learn the set of trees {*f*_*i*_}, the regularized objective function is defined as follows [70]:

2 $$\begin{array}{@{}rcl@{}}  L(\phi) = \sum\limits_{i}l({\hat y}_{i},y_{i}) + \sum\limits_{k} \Omega (f_{k}) \end{array} $$

where *l* is a differentiable convex loss function that is used to calculate the difference between the prediction ${\hat y}_{i}$ and target *y*_*i*_. To avoid the overfitting, the second term *Ω* is used to control the complexity of the model. This regularized function can penalize the complicated models. Finally, the model with simple and predictive functions can be selected.

Since this model includes functions as parameters, it can not use traditional optimization methods in the Euclidean space to establish it. Instead, a new tree *f*_*t*_ is added to the ensemble, which optimizes the objective function and is searched from the functional space *F* at each iteration *t*. The process is defined as follows:

3 $$ \begin{aligned}  L^{(t)} &= \sum\limits_{i=1}^{n} l\left(y_{i},{\hat y}_{i}^{(t)}\right) + \sum\limits_{i=1}^{t} \Omega (f_{i}) \\ &= \sum\limits_{i=1}^{n} l\left(\left(y_{i},{\hat y}_{i}^{(t-1)}\right)+f_{t}(x_{i})\right) + \sum\limits_{i=1}^{t} \Omega (f_{i}) \end{aligned}  $$

where ${\hat y}_{i}^{(t)}$ is the prediction of the *i*−*th* instance at the *t*−*th* iteration. The model finds *f*_*t*_ to optimize the above objective function.

Equation (3) is still hard to optimize in the general setting, so the second order Taylor expansion is used to approximate the objective function as follows:

4 $$ \begin{aligned}  L^{(t)} \simeq \sum\limits_{i=1}^{n} \left[l\left(\left(y_{i},{\hat y}_{i}^{(t-1)}\right) + g_{i}f_{t}(x_{i})\right.\right.\\ +\left.\left.\frac{1}{2}h_{i}{{f_{t}}{^{2}}}(x_{i})\right)\right] + \sum\limits_{i=1}^{t} \Omega (f_{i}) \end{aligned}  $$

where $g_{i}=\partial _{\hat {y}_{i}^{(t-1)}} l\left (y_{i}, {\hat {y}_{i}^{(t-1)}}\right)$ and $h_{i}= {\partial _{\hat {y}_{i}^{(t-1)}}}^{2} l\left (y_{i}, {\hat {y}_{i}^{(t-1)}}\right)$. By removing the terms independent of *f*_*t*_(*x*_*i*_), the following approximate objective function at step *t* can be obtained:

5 $$ \begin{aligned}  \overline{L}^{(t)} = \sum\limits_{i=1}^{n} \left[g_{i}f_{t}(x_{i}) + \frac{1}{2}h_{i}{{f_{t}}{^{2}}}(x_{i}))\right] + \sum\limits_{i=1}^{t} \Omega (f_{i}) \end{aligned}  $$

A gradient boosting algorithm iteratively adds functions that optimizes $\overline {L}^{(t)}$ for a number of user-specified iterations.

In order to learn the function *f*_*t*_ in each step, the mapping *q*:*R*^*d*^→{1,2,...,*T*} is defined to map the input to the index of the region. The function is defined as follow:

6 $$  f_{t}{(X)} = w_{q(X)}  $$

where *w* is a vector of scores in each region and *q* represents the decision tree structure. Furthermore, the function complexity was defined as follow:

7 $$  \Omega (f_{t}) = \gamma T + \frac{1}{2} \lambda \sum\limits_{j=1}^{T} {w_{j}^{2}}  $$

where *T* is the number of trees. The parameters *γ* and *λ* are used to make a balance. ${w_{j}^{2}}$ is the prediction score for data corresponding to the *j*−*th* leaf from *f*_*t*_.

Then Eq(5) can be rewritten as follow:

8 $$  \overline{L}^{(t)} = \sum\limits_{j=1}^{T}\left[\left(\sum\limits_{i \in I_{j}}g_{i}\right)w_{j} + \frac{1}{2}\left(\sum\limits_{i \in I_{j}}h_{i} + \lambda \right){w_{j}^{2}}\right] + \gamma T  $$

where *I*_*j*_={*i*|*q*(*x*_*i*_)=*j*} is defined as the instance set of region *j*. When *q*(*x*) is fixed, the optimal weight ${w_{j}^{*}}$ of region *j* can be calculated as follows:

9 $$  {w_{j}^{*}} = - \frac{{\sum\nolimits}_{i \in I_{j}}g_{i}}{{\sum\nolimits}_{i \in I_{j}}h_{i} + \lambda}  $$

The optimal objective value is calculated as follow:

10 $$  \overline{L}^{(t)}(q) = - \frac {1}{2} \sum\limits_{j=1}^{T} \frac{({\sum\nolimits}_{i \in I_{j}}g_{i})^{2}}{{\sum\nolimits}_{i \in I_{j}}h_{i} + \lambda} + \gamma T  $$

Equation (10) is used to score the region partition specified by *q*. It also can find a good structure according to the previous reference [70]. Since there can be infinitely many possible candidates of the tree structure, it applied a greedy algorithm in practice [70]. The one step of the algorithm was that splitting a leaf into two leaves. In each round, it greedily enumerated the features and split the feature that gives the maximum reduction calculated by Eq. (10). The main feature of this model is the explicit regularization term which prevents the model from overfitting. The detail of this model can be found in Chen et al. [67].

## Results

### Performance evaluation

In order to assess the prediction performance of our method and other computing methods, we conduct the 5CV based on the same benchmark dataset. The competing methods include miES [51], GaussianNB [71] and SVM [72, 73]. The benchmark dataset is downloaded from miES. In each round of the 5CV, we divide the essential miRNAs and non-essential miRNAs into the 5 sets, 4 of which are used to train the model while the left one is used as the testing set. We repeat the 5CV 50 times in this study.

In addition, the AUC value is used to measure the prediction performance of computational methods. The ROCs are drawn with TPR (true positive rate) with respect to FPR (false positive rate) values. TPR is the fraction of essential miRNAs that are correctly predicted, while FPR is the fraction of non-essential miRNAs that are incorrectly predicted. Furthermore, the F-measure and ACC are also used to evaluate the prediction performance of computational methods. The F-measure is calculated from the harmonic mean of precision (*P*) and recall (*R*)(*F*=2∗*P*∗*R*/(*P*+*R*)).

### Comparison with other competing methods

**Fig. 1:**
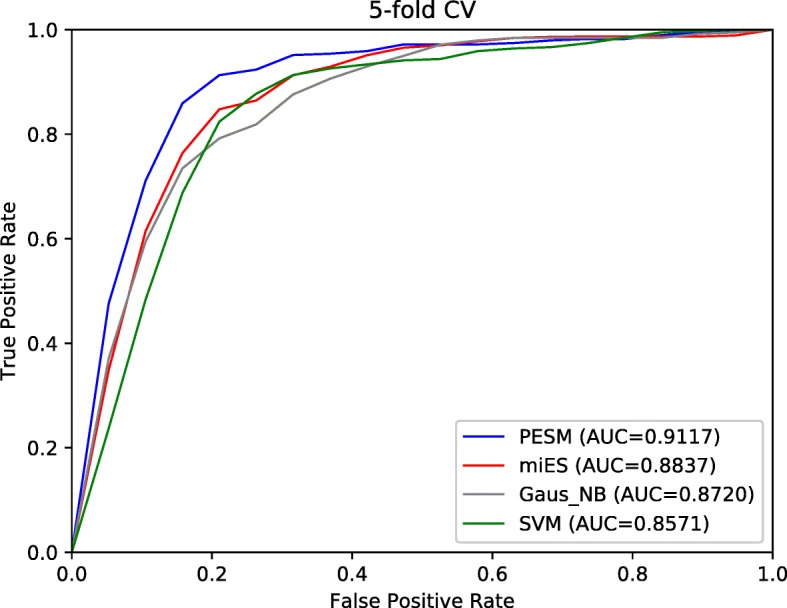
The ROC plot of the four computational methods with on the 5-fold cross validation

In this study, we compare our method to other three competing methods which include miES, GaussianNB and SVM. MiES was a computational method for miRNA essentiality prediction, which only uses sequence features of known essential miRNAs. In addition, GaussianNB and SVM are the typical classification models. Figure 1 plots the ROC curve and shows the AUC values of four computational methods. In terms of AUC, our method obtains the best prediction performance as its AUC value is 0.9117, compared with other methods (miES: 0.8837, GaussianNB: 0.8720 and SVM: 0.8571).

In addition, Table 2 shows the ACC and F-measure values of four methods with the 5CV validation. We can see from Table 2 that our method obtains the best prediction performance (ACC:0.8516 and F-mearsure:0.8572), compared with other methods (miES (ACC:0.8263 and F-mearsure:0.8326), GaussianNB (ACC:0.8000 and F-mearsure:0.8093) and SVM (ACC:0.8206 and F-mearsure:0.8271)).

**Table 2:** The ACC and F-measure values of four computational methods with on the 5-fold cross validation

Method	ACC	F-measure
PESM	0.8516	0.8572
miES	0.8263	0.8326
GaussianNB	0.8000	0.8093
SVM	0.8206	0.8271

## Relative importance of the features

**Fig. 2:**
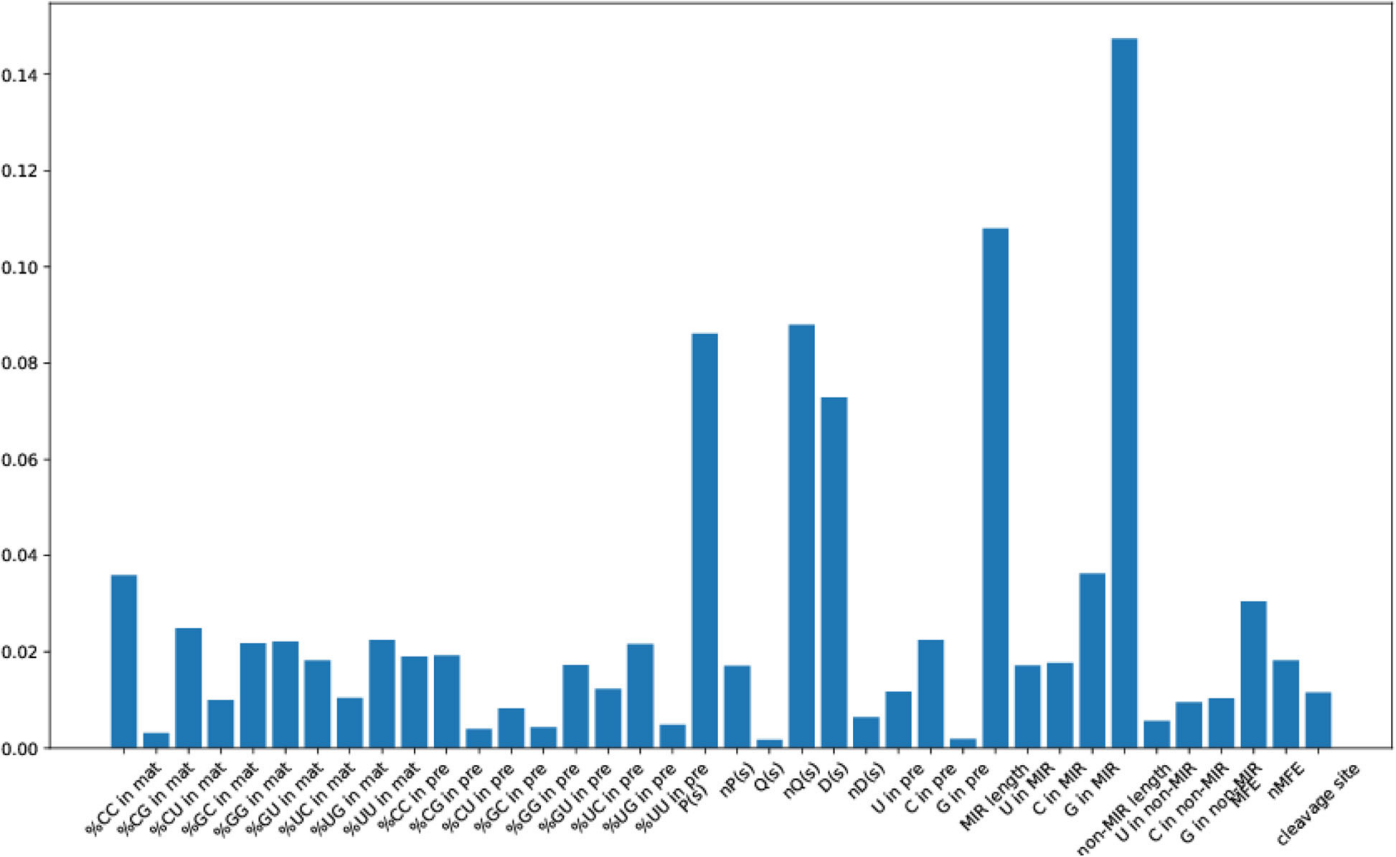
The relative importance of all 38 features. pre-miR means pre-miRNA; MIR means mature miRNA; non-MIR means non-mature-miRNA

In order to demonstrate the newly added features in the prediction method, we further analyze the relative importance of all 38 features. Figure 2 plots the relative importance of the features, which is computed by the XGBoost package. We can see from Fig. 2 that 4 newly added features are ranked top 10 based on the relative importance, which include *%CC* in mat, *P*(*s*),*nQ*(*s*) and *D*(*s*). These 4 added features rank 6, 4, 3 and 5, respectively. It also demonstrates that the newly added features can reflect the intrinsic characteristics of miRNAs and help improve the performance of predicting essential miRNAs.

## Parameter analysis for *γ*,*λ*, *K* and *T*

In this study, we analyze four parameters, including the regularization terms on the number of regions (*γ*), on the sum of squared scores (*λ*), the maximum depth of regression trees (*K*) and the number of trees (*T*). The default values of *γ*,*λ*, *K* and *T* are 0, 0.1, 6 and 1000, respectively. We conduct the 5CV to evaluate the prediction performance of PESM. In addition, one of four parameters is analyzing while the other three parameters are set to be the default values.

The default value of *γ* is 0 in the XGBoost package. We also compute the prediction performance of PESM with the parameter *γ* in the set 0, 0.1, 0.2 according to reference [70]. The AUC values of our method are 0.9117, 0.9133 and 0.9100. In this study, we set the value of parameter *γ* to 0 based on our experiment results and the default value in the XGBoost package.

We evaluate the prediction performance of PESM when parameter *λ* ranges from 0.25 to 2.0 with the increment of 0.25. We can see from Table 3 that PESM can achieve the best prediction performance when it is set to 1.0 which is also the default value of XGBoost package. Therefore, we set *λ* to 1.0 in this study.

**Table 3:** The prediction performances of PESM with different settings of *λ*

*λ*	0.25	0.50	0.75	1.0
AUC	0.9116	0.9116	0.9116	0.9117
*λ*	1.25	1.50	1.75	2.0
AUC	0.9083	0.9041	0.9025	0.9041

Furthermore, in the XGBoost package, the default value of parameter *K* is 6. Table 4 describes the AUC values obtained by PESM when *K* ranges from 3 to 9. We can see from Table 4 that our method can obtain the best prediction performance when *K* is set to be 7, and obtain reliable prediction performances when *K* ranges from 5 to 7. Therefore, by considering the default value in the XGBoost package and our experiments results, we set *K* to 6 in this study.

**Table 4:** The prediction performances of PESM with different settings of *K*

*K*	3	4	5	6	7	8	9
AUC	0.9066	0.9067	0.9116	0.9117	0.9133	0.9058	0.9053

Finally, Table 5 shows the prediction performance of PESM when the tree number *T* is set to 100, 500, 1000, 1500, 2000. We can see from Table 5 that PESM obtain the reliable prediction performance when *T* is selected from one of set 1000, 1500, 2000. Therefore, we also set the default value of *T* to 1000 in this study.

**Table 5:** The prediction performances of PESM with different settings of *T*

*T*	100	500	1000	1500	2000
AUC	0.8958	0.9068	0.9117	0.9141	0.9113

## Discussion

With the development of biotechnology, studies have shown that miRNAs participate in many biological processes, such as cell growth, cell death and so on. Furthermore, miRNAs also play important roles in human diseases, especially the complex diseases, such as cancer. Therefore, the study of miRNA and disease associations has become a main research topic in bioinformatics. Based on the more systematic understanding of miRNAs, studies further demonstrate that some miRNA molecules are essential to the disease development. The essential miRNAs are necessary to manifest principles of disease mechanisms. Therefore, identifying the essential miRNAs is very appealing.

## Conclusion

In this study, we have developed a computational method (PESM) to predict the essentiality of miRNAs. PESM integrates the 38 sequence and structural features of miRNAs. Then it further uses the gradient boosting machines to compute the predicted scores of essential miRNAs. The experiment results with the 5-fold cross validation show that the prediction performance of PESM is superior to other competing methods, including the state-of-art method miES. Finally, we have analyzed the relative importance of all features by the XGBoost package, and the results demonstrate that the newly added features can further improve the prediction performances.

Although our method can effectively predict the essential miRNAs and non-essential miRNAs, its limits should be addressed in the future. First, the non-essential miRNAs in the current benchmark dataset are randomly selected. Second, the more features of miRNAs also should be designed, such as topological features of miRNAs. Finally, other similarity-based methods [74], collaborative metric learning methods [75] and deep learning methods [76, 77] should be adopted. We would provide a more effective computational method to predict essential miRNAs by addressing above limitations in the future.

## Data Availability

The datasets and source codes are available at https://github.com/bioinfomaticsCSU/PESM.

## Abbreviations

5CV: 10-fold cross validation
ACC: Accuracy
AUC: Area under the receiver operating curve
FPR: False positive rate
GaussianNB: Gaussian Naive Bayes
MFE: minimum free energy
SVM: Support vector machine
TPR: True positive rate
